# Does posterior configuration have similar strength as parallel configuration for treating comminuted distal humerus fractures? A cadaveric biomechanical study

**DOI:** 10.1186/s12891-021-04302-9

**Published:** 2021-05-14

**Authors:** Chien-An Shih, Fa-Chuan Kuan, Kai-Lan Hsu, Chih-Kai Hong, Cheng-Li Lin, Ming-Long Yeh, Wei-Ren Su

**Affiliations:** 1 Department of Biomedical Engineering, National Cheng Kung University, Tainan, Taiwan; 2 Department of Orthopaedic Surgery, National Cheng Kung University Hospital, College of Medicine, National Cheng Kung University, Tainan, Taiwan; 3 Medical Device R & D Core Laboratory, National Cheng Kung University Hospital, Tainan, Taiwan; 4 Department of Orthopedics, National Cheng Kung University Hospital Dou-Liou Branch, Tainan, Taiwan; 5 Medical Device Innovation Center, National Cheng Kung University, Tainan, Taiwan

**Keywords:** Biomechanical study, Distal humeral fracture, Fracture fixation, Comminuted fracture, Parallel plating, Posterior plating

## Abstract

**Background:**

The posterior plating technique could be used as a clinical alternative to parallel plating for treating comminuted distal humerus fractures (DHFs) successfully with good clinical results. However, the biomechanical characteristics for posterior fixation are still unclear. The purpose of this study is to evaluate the biomechanical properties of the posterior fixation and to make comparisons between the parallel and the posterior fixation systems.

**Materials and methods:**

We performed a cadaveric biomechanical testing with two posterior plating systems (a posterior two plating and a single posterior pre-contoured Y plating system) and one parallel two plating system to treat AO/OTA type-C2.3 DHFs. Among three groups, we compared construct stiffness, failure strength, and intercondylar width changes after 5000-cycle fatigue loading and failure loads and failure modes after destructive tests in both the axial compression and (sagittal) posterior bending directions. The correlations between construct failure loads and bone marrow density (BMD) were also compared.

**Results:**

In axial direction, there were no significant differences in the stiffness and failure load between the posterior and the parallel constructs. However, in sagittal direction, the two-plate groups (posterior two plating and parallel plating group) had significant higher stiffness and failure loads than the one-plate group (single posterior Y plating). There was no fixation failure after 5000-cyclic loading in both directions for all groups. Positive correlation was noted between BMD and failure loads on parallel fixation.

**Conclusions:**

We found that when using two plates for treating comminuted DHFs, there were no significant differences in terms of most biomechanical measurements between posterior and parallel fixation. However, the single pre-contoured posterior Y plate construct was biomechanically weaker in the sagittal plane than the parallel and the posterior two-plate constructs, although there was no fixation failure after the fatigue test for all groups regardless of the fixation methods.

**Level of evidence:**

Biomechanical study

**Supplementary Information:**

The online version contains supplementary material available at 10.1186/s12891-021-04302-9.

## Introduction

Comminuted distal humerus fractures (DHFs) comprise one of the most challenging treatment situations in elbow injuries [1]. The surgical treatment principle for a distal humerus fracture is bicolumnar fixation with plate and screws [2, 3]. Post-operative complications due to inadequate fixation, such as non-union, mal-union and loss of reduction, can affect clinical outcomes [4–6].

Current popular fixation methods for comminuted DHFs are double plating in either parallel or orthogonal direction, which have been studied widely in both clinical and biomechanical aspects [7–9]. However, orthogonal plating is reported to have inadequate fixation in cases with osteoporotic and comminuted fractures, which may lead to fixation failure or non-unions [10]. Recently, parallel constructs had gained more surgical popularity for treating comminuted DHFs due to the superiority over orthogonal constructs based on recent biomechanical studies [8, 11–13] and a biomechanical meta-analysis [9]. However, the main disadvantage of the parallel fixation is the wide soft tissue dissection [9, 12].

Clinically, the posterior bicolumnar fixation technique, including a single posterior Y plate (YP) system [14, 15] and a posterior two plate (PTP) system [16], is an alternative to the parallel fixation in treating comminuted distal humerus fractures. Studies on posterior fixations showed that it could have good clinical results, high union rates, and low complication rates [14–16], and may not require as wide surgical dissection as the parallel approach. To our knowledge, although there was a biomechanical comparative study between posterior and orthogonal configurations in treating a-type (supracondylar) DHFs [17], there were none between posterior and parallel configurations in treating c-type comminuted DHFs.

In this study, we aim to make comparisons between the posterior and the parallel locking-plate fixation systems and to evaluate the effects of plate numbers (one versus two) on the strength of fixation. Our hypotheses are that (1) the posterior two-plate fixation system may provide as much biomechanical stabilities as parallel fixation, and that (2) the single posterior Y plate system may be biomechanically weaker than both the posterior and parallel two-plate fixation system.

## Materials and methods

### Specimen preparation

Twenty-seven humeri specimens were obtained from MedCure© (Portland, OR) anatomic tissue bank. The use the specimen was based on Medcure guidelines. The specimens were harvested from 11 female and 16 male fresh-frozen cadavers (mean age at death, 76 (mean) ± 11 (SD) years). We stored the humeri at − 20 °C before mechanical testing preparation. Bone mineral density (BMD) was evaluated using dual-energy X-ray absorptiometry (DXA) (GE medical system, Lunar Prodigy, Madison, WI, USA). The humeri were randomly allocated into two cohosts for different directional loading (axial: 12 humeri; sagittal: 15 humeri). The humeri in each cohost was then divided into three groups based on implant configurations (axial: 4 specimens/group, sagittal: 5 specimens/group).

In each specimen, we used a 0.5 mm-wide bone saw to simulate Arbeitsgemeinschaft für Osteosynthesefragen (AO)/Orthopaedic Trauma Association (OTA) type 13-C2.3 fractures with the creation of a 10-mm wide gap above the top of the olecranon fossa to simulate supracondylar comminution [7, 13] and inter-condylar osteotomy at the deepest point of the tracheal groove [7, 8, 12, 18].

### Implant osteosynthesis, plotting, and tracking system preparation

**Fig. 1:**
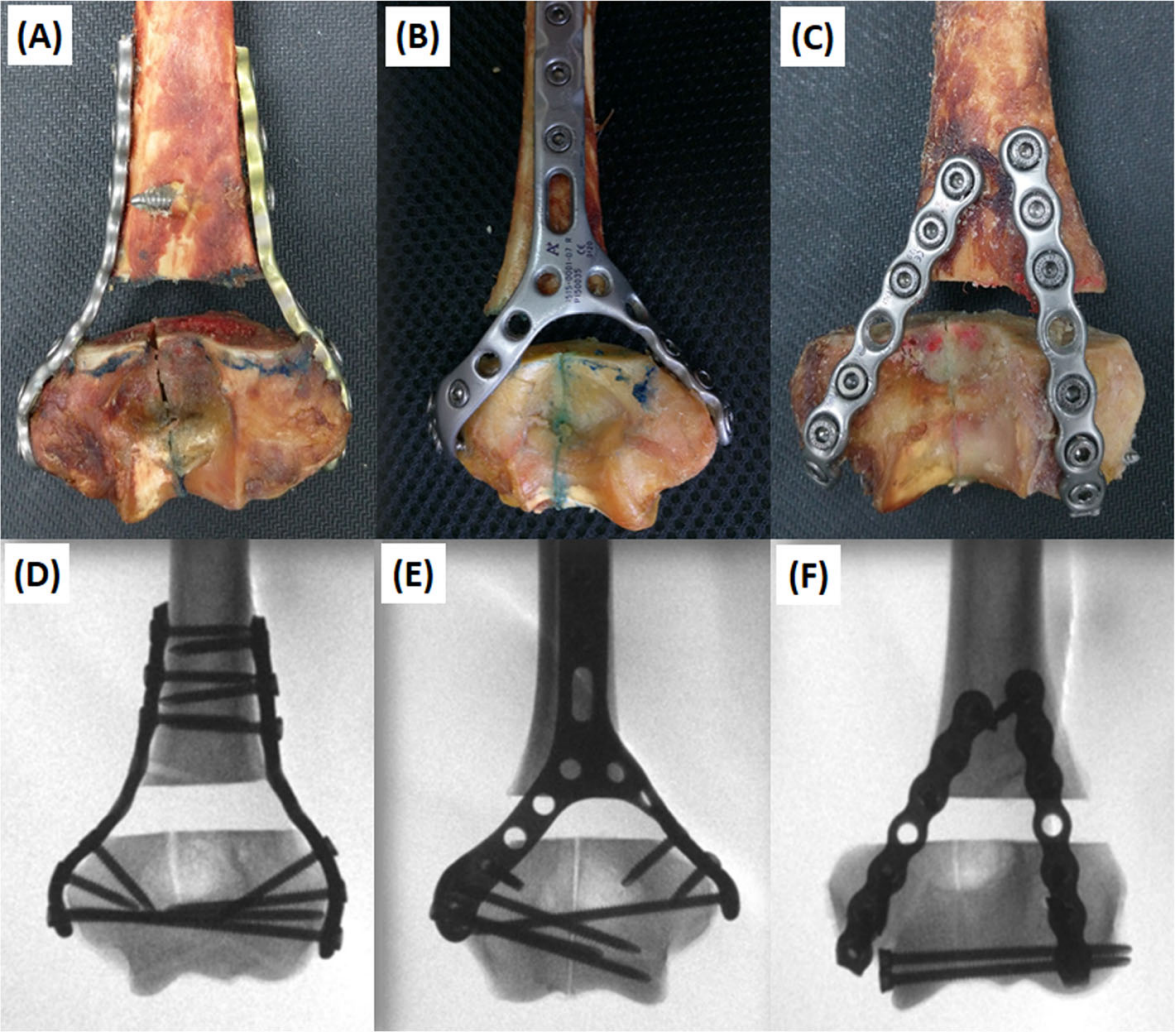
Complete plating and radiographic confirmation for the three different testing groups. **a** and **d** parallel plating; **b** and **e** Y plating; **c** and **f** posterior two plating

A single senior orthopedic surgeon performed all of the procedures. In each cohort, the specimens were fixed with either parallel, posterior Y, or a posterior two locking plate (LP) system (Aplus Reconstruction or Distal Posterior Humerus Anatomical Arch-Y Locking Plate System; Ti-6Al-4 V titanium alloy plate; APlus Biotechnology Co., Ltd., New Taipei City, Taiwan, Republic of China). We predrilled all specimens to guarantee anatomic reduction. Osteotomies were then performed after pre-drilling and plate removal. The humeri in parallel plate (PP) group were fixed with a medial and a lateral reconstruction LP (Fig. 1a). The single Y plate (YP) group was treated with a single posterior pre-contoured LP (Fig. 1b), which permits both distal screw interdigitated fixation and bicolumnar fixation. The application of medial and lateral plate arms from opposing parallel directions of the Y plate provides fixation to the distal humerus fragment only (Fig. 1b). The posterior two plate (PTP) group was treated with two posterior reconstruction LPs with the addition of two intercondylar screw fixation (Fig. 1c).

All specimens were then assembled, and the proximal ends were plotted in a 6 cm cylinder tube filled with a commercially available industrial concrete. Then, each humerus was mounted using a Fastrak freedom magnetic tracking system (Polhemus, Colchester, VT, USA). We placed three trackers on each segment using commercially available quick-bond materials with two non-mental screws fixation. Three trackers were used for each specimen: two trackers on the distal medial and lateral segments placed 5-mm from the intercondylar fracture line and 5-mm distal to the fracture gap and one tracker on the shaft segment set in the long axis of the shaft 5-mm proximal to the fracture gap.

### Biomechanical testing and analysis

**Fig. 2:**
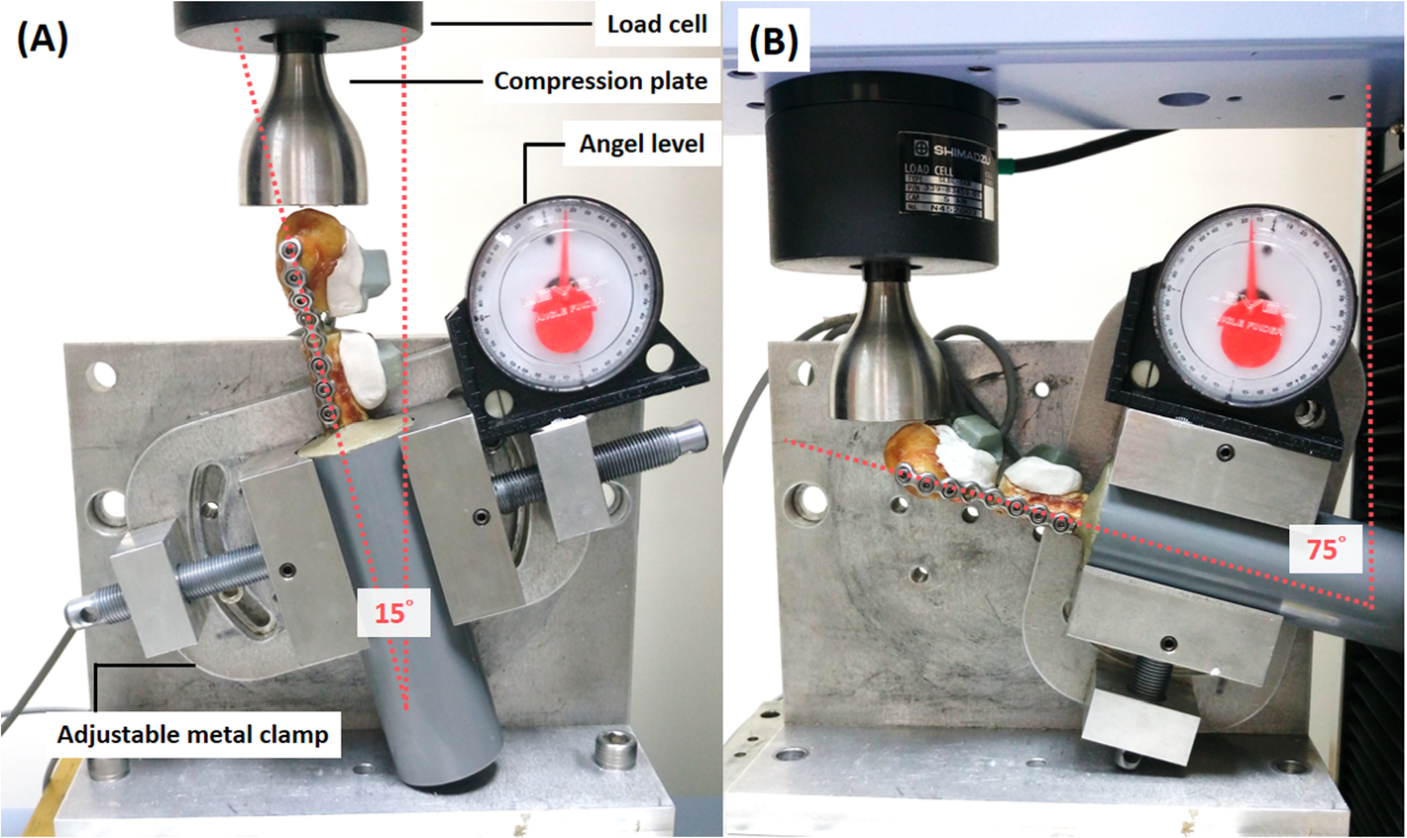
Biomechanical testing setups of distal humerus fractures. **a** axial loading, **b** posterior bending: adjustable metal clamp, load cell, angle device and motion trackers

The test setups using a testing machine with a 5kN load cell (AG-X; Shimadzu Corp., Tokyo, Japan) were modified from a prior protocol [19]. Each specimen was loaded with cylinder in an adjustable metal clamp with 15° flexion [19] for the axial compressive loading (Fig. 2a) and 75° flexion [18–20] for the anterior-posterior bending load application in sagittal plane (Fig. 2b), representing the extension and flexion loading of arm. A 6-cm diameter flat stainless-steel compression plate was used for load application over the distal end of entire condyle in each specimen (Fig. 2a). All cadaveric humeri underwent a nondestructive axial compression or posterior bending test, following by a cyclic loading test and a destructive test.

The mechanical test procedure was modified from a previous biomechanical study [21]. First, each specimen was preloaded to 50 N at a speed of 1 mm/min for 5 cycles. Construct stiffness was calculated over a range from 20 to 40 N in the axial compression and posterior bending tests. Next, we performed cyclic-fatigue loading in either the axial compression or posterior bending direction for all humeri (valley/peak force: 50/200 N; 0.1 Hz for 5000 cycles). Construct stiffness after cyclic loading was calculated from 50 to 200 N at a speed of 1 mm/min via a separate ramped test. Finally, all humeri underwent a destructive test at a 1 mm/s rate. We stopped the testing when construct failure was noted based on a previous study [11], including plastic deformation (an axial or sagittal displacement > 1 mm) or the occurrence of mechanical failure /implant loosening (bone implant fractures, implant breakage, and bone-screw interface loosening).

Failure stiffness in the load-to-failure test was calculated based on the linear part in the load-displacement curve. Elastic limit and load to failure were also recorded. Failure modes were analyzed and compared for each group. Three Fastrak freedom magnetic trackers were used to detect and calculate the changes in the intercondylar width and the local medial and lateral column interfragmental displacements in relative to the shaft column after the 5000-cycle fatigue test.

### Statistical analyses

For the group comparison between different biomechanical parameters after cyclic loading, we performed a Kruskal-Wallis test for post-cyclic stiffness, elastic limit, failure stiffness, failure load, lateral and medial column interfragmental displacements and changes of intercondylar width and then use a Mann-Whitney U test as a post hoc test (Supplementary Table 1). A Spearman correlation test was used to make correlations between failure loads and BMD. Significance was set at *p* < 0.05. MedCalc 14 for Windows (Medcalc Software, Ostend, Belgium) was used for all analyses.

#### Post hoc power analyses

Post-hoc power analyses were performed with G*Power (version 3.1.9.2; http://www.gpower.hhu.de; Heinrich Heine-University of Dusseldorf, Dusseldorf, Germany) to calculate the achieved power. An alpha equal to 0.05 was given for the parameters after cyclic loading.

## Results

### Biomechanical properties

#### Stiffness, elastic limit and failure load

Stiffness, Elastic limit and failure load in axial and posterior bending (sagittal) directions after 5000-cycle loading were compared (Table 1), and the post-hoc Mann-Whitney tests for pairwise comparisons were summarized (Supplementary Table 1).

**Table 1:** Biomechanical measurements (stiffness, elastic limit, failure stiffness, failure load) and the distal medial and lateral column interfragmental displacements without loading force in axial loading and posterior bending directions after the 5000-cycle fatigue test for the parallel, Y, and posterior two plating group

	Parallel plating	Y plating	Posterior two plating	*P*-value (Kruskal-Wallistest)
Axial loading				
Stiffness (50-200N)(N/mm	590.20 ± 125.93	632.35 ± 182.44	607.50 ± 91.91	0.981
Elastic limit (N)	377.36 ± 115.34	502.96 ± 214.44	357.49 ± 132.23	0.491
Failure stiffness (N/mm)	500.73 ± 165.18	460.13 ± 33.94	517.27 ± 105.15	0.595
Failure load (N)	1795.87 ± 413.66	1181.36 ± 249.11	1309.14 ± 274.26	0.116
Lateral column displacement (mm)	1.60 ± 1.64	1.38 ± 0.89	1.62 ± 0.85	0.594
Medial column displacement (mm)	1.88 ± 2.36	2.28 ± 0.79	0.80 ± 0.58	0.155
Posterior bending				
Stiffness (50-200N)(N/mm)	181.61 ± 58.81	34.76 ± 11.04^†^	309.87 ± 97.99	0.004
Elastic limit (N)	185.26 ± 36.42	157.10 ± 22.11	171.12 ± 56.12	0.566
Failure stiffness (N/mm)	80.38 ± 29.49	18.05 ± 4.88^†^	174.80 ± 59.20^‡^	0.002
Failure load (N)	568.86 ± 202.13	283.31 ± 38.16^†^	594.52 ± 122.95	0.009
Lateral column displacement (mm)	1.83 ± 0.81	10.73 ± 6.04^‡^	2.18 ± 0.73	0.008
Medial column displacement (mm)	2.45 ± 2.55	15.36 ± 7.59^‡^	1.63 ± 0.59	0.009

^†^Significantly the lowest with post-hoc test (Mann-Whitney U-test; supplementary table 1) for pairwise comparisons, *p* < 0.05

^‡^Significantly the highest with post-hoc test (Mann-Whitney U-test; supplementary table 1) for pairwise comparisons, *p* < 0.05

In axial compression loading, there were no significant between-group differences regarding posterior or parallel configuration for construct stiffness, failure stiffness, elastic limit and failure loads.

However, in terms of posterior bending, either parallel (PP) or posterior (PTP) system with two plating had significantly higher construct stiffness (*p* = 0.004, post hoc achieved power = 99.9%), failures stiffness (*p* = 0.002, post hoc achieved power = 99.9%), and failure loads (*p* = 0.009, post hoc achieved power = 88.3%) than the single posterior Y plating group after 5000 cyclic loading. When comparing the two-plate construct groups, the posterior two plate (PTP) system had significantly higher failure stiffness than the parallel plate (PP) one (*p* = 0.016, post hoc achieved power = 88.1%). However, there were no significant between-group differences regarding other parameters (stiffness and failure loads) for PTP and PP groups.

#### Evaluation of local interfragmental displacements of medial and lateral columns after 5000-cycle loading

Local interfragmental displacements of the medial and lateral columns after the 5000-cylcle fatigue test were compared (Table 1), and the post-hoc Mann-Whitney tests for pairwise comparisons were also summarized (Supplementary Table 1).

It can be seen that in terms of axial compressive loading, there were no significant among-group differences in the local lateral or medial interfragmental displacement of any of the column. However, in posterior bending, the single plating (YP) group had significantly more interfragmental lateral column displacements than the PP group (*p* = 0.008, post hoc achieved power = 89.3%) and the PTP group (*p* = 0.008, post hoc achieved power = 87.2%), and significantly more interfragmental medial column displacements than the PP group (*p* = 0.008, post hoc achieved power = 93.8%) and the PTP group (*p* = 0.008, post hoc achieved power = 97.2%).

#### Evaluation of intercondylar width changes and fixation stability after 5000-cycle loading

We measured the intercondylar width using the Fastrak freedom magnetic tracker on the medial and lateral columns. Then, the intercondylar width changes without loading force (0 N) before and after the fatigue test were calculated and compared (Table 2). The post-hoc Mann-Whitney tests for pairwise comparisons were also summarized (Supplementary Table 1). The results showed that there was no intercondylar segment separation or screw loosening in any of the group regardless of the fixation method after 5000-cycle loading. For both loading directions, there were also no within-group differences in changes of intercondylar width for both posterior and parallel systems after the 5000-cycle fatigue test.

**Table 2:** Measurements of the intercondylar width changes (Δ) without loading force (0N) before and after the 5000-cycle fatigue test for the parallel, Y, and posterior two plating constructs

		Parallel plating	Y plating	Posterior two plating	*P*-value (Kruskal-Wallistest)
Axial loading	ΔIntercondylar width (mm) (loading force: 0N)	0.40 ± 0.26	0.26 ± 0.31	0.78 ± 0.63	0.246
Posterior bending	ΔIntercondylar width (mm) (loading force: 0N)	0.90 ± 0.65	1.98 ± 1.37	0.62 ± 0.55	0.151

### Failure modes

**Fig. 3:**
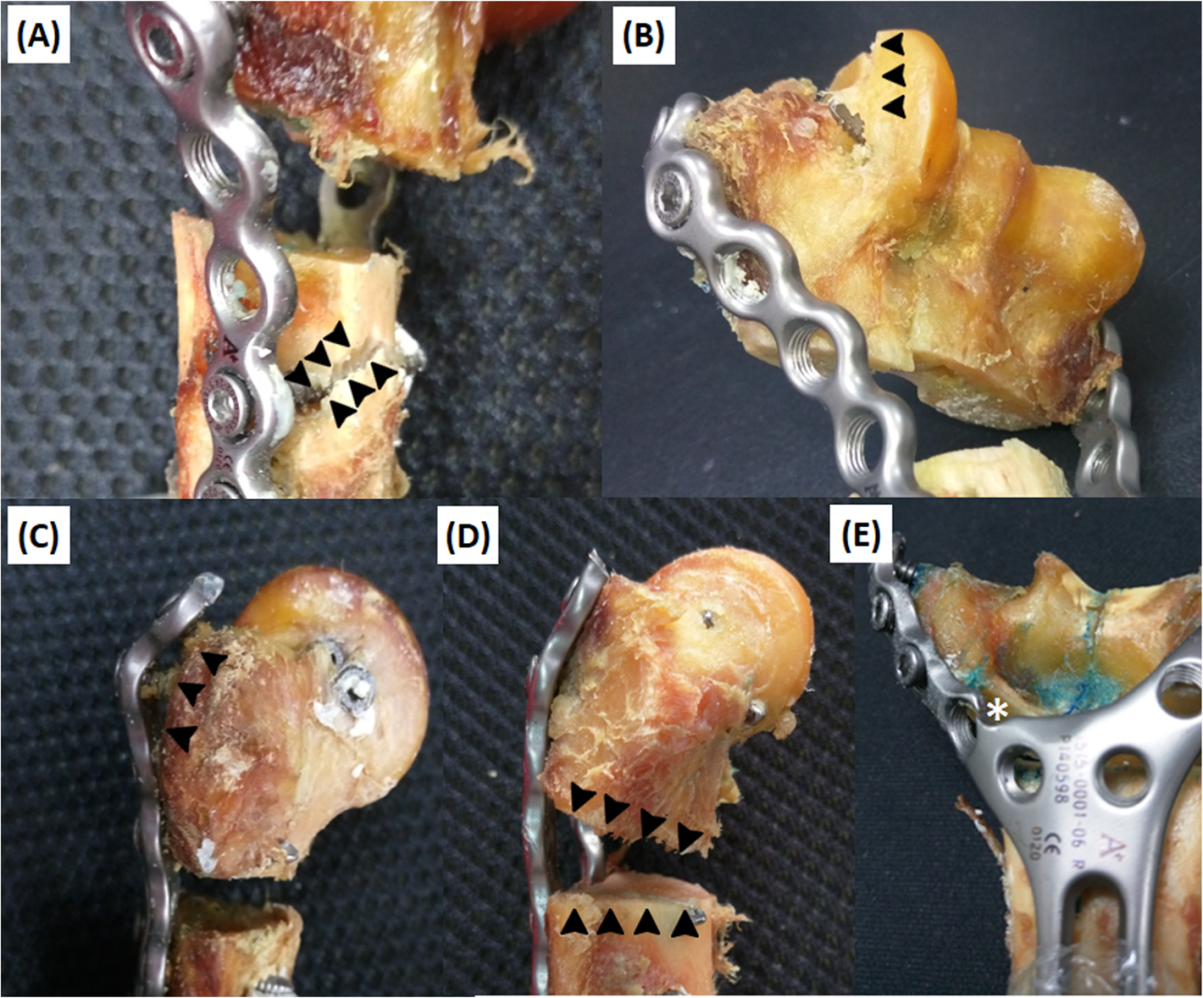
Failure mode illustrations: **a** proximal bone-screw loosening, **b** distal bone-screw loosening, **c** distal bone fracture, **d** plate plastic deformation, and **e** implant breakage

Failure modes for different fixations were classified into three categories [11] (Table 3): (1) proximal bone-screw interface loosening (Fig. 3a), (2) distal bone-screw interface loosening (Fig. 3b and c), and (3) plate plastic deformation (Fig. 3d). Regardless of the loading direction, the parallel group failed primarily due to proximal or distal bone-screw interface loosening (Axial: 100%; Sagittal 100%), while the posterior groups failed primarily due to plate plastic deformation (Axial: 75%; Sagittal 80%).

**Table 3:** Failure modes for the parallel, Y, and posterior two plating configurations in axial loading and posterior bending directions

	Axial loading (*n* = 12)			Posterior bending (*n* = 15)		
	Parallel plating (*n* = 4)	Y plating (*n* = 4)	Posterior two plating (*n* = 4)	Parallel plating (*n* = 5)	Y plating (*n* = 5)	Posterior two plating (*n* = 5)
(1) Proximal bone-screw interface loosening	0/4	0/4	0/4	2/5 (40%)	0	0
(2) Distal bone-screw interface loosening	4/4 (100%)	0/4	2/4 (50%)	3/5 (60%)	0	2/5 (40%)
(3) Plastic deformation	0/4	4/4 (100%)	2/4 (50%)	0	5/5 (100%)	3/5 (60%)

However, there was a slight difference between the two posterior systems. The single posterior Y plate group (YP) failed entirely due to plate plastic deformation (Axial: 100%; Sagittal 100%). In contract, some of the specimens in the posterior two-plate group (PTP) failed due to distal bone-screw loosening (Axial: 50%; Sagittal 40%), and the others failed due to plastic deformation (Axial: 50%; Sagittal 60%). One plate in the YP group, representing as 1.6% for all specimens, failed due to implant breakage during axial loading after plastic deformation (Fig. 3e).

### Failure load and BMD

**Fig. 4:**
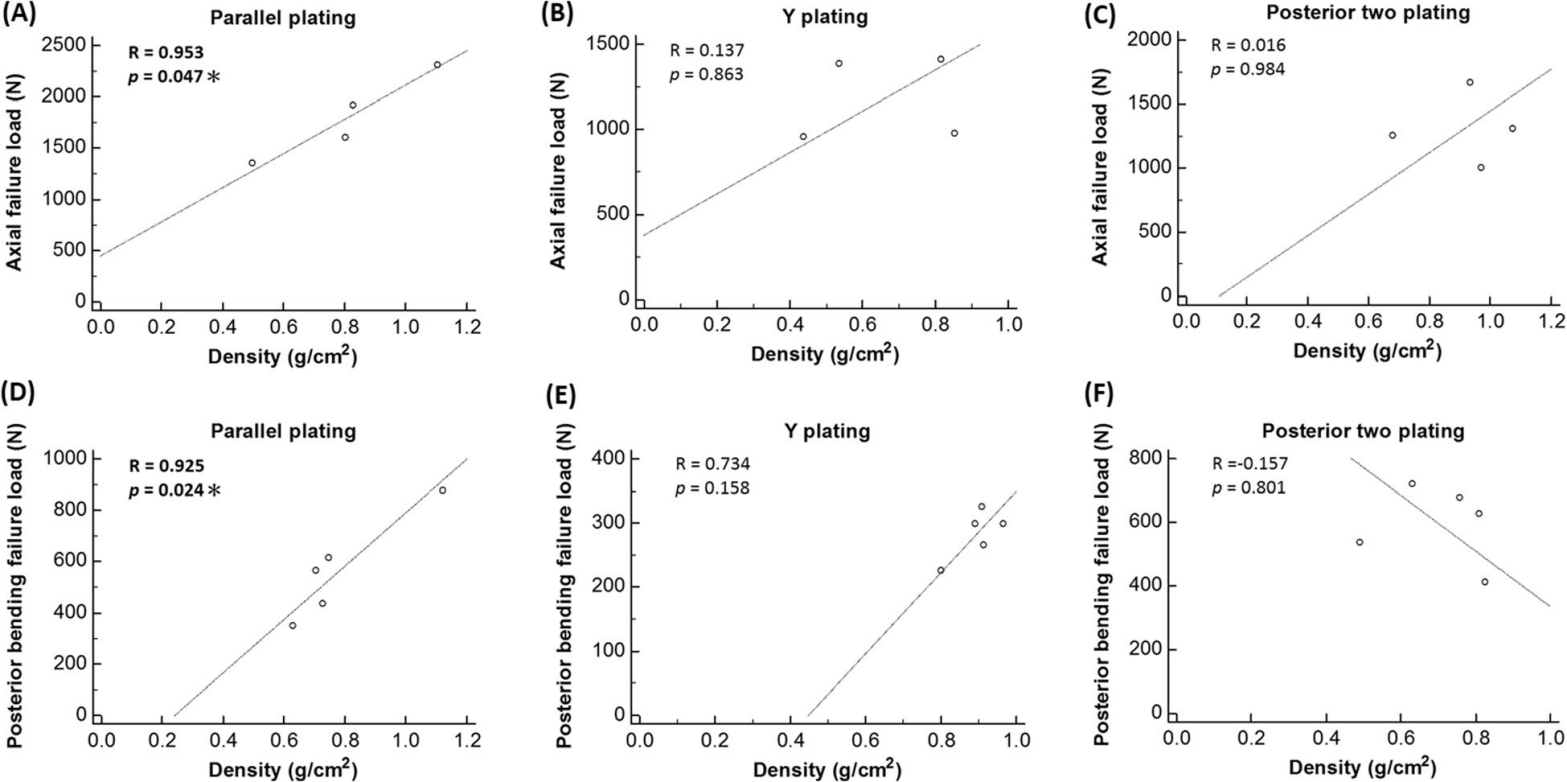
Pearson correlation coefficient (*R*) and *P*-value (*p*) between failure load and the three experimental groups: **a** axial failure load and parallel plating group, **b** axial failure load and Y plating group, **c** axial failure load and posterior two plating group; **d** posterior bending failure load and parallel plating group, **e** posterior bending failure load and Y plating group, **f** posterior bending failure load and posterior two plating group

The failure loads in the parallel system (PP) displayed significantly positive correlations with BMD in both loading directions (Axial loading: *R* = 0.953, *p* = 0.047, Fig. 4a; Posterior bending, *R* = 0.925, *p* = 0.024, Fig. 4d). In contrast, the failure loads in the posterior system did not correlate with BMD in either the YP (Fig. 4b and e) or the PTP group (Fig. 4c and f).

## Discussion

In this study, we compared the construct stiffness, intercondylar width changes, failure stiffness, elastic limit, and failure load for posterior and parallel fixation systems. With regards to axial compression loading, there were no significant differences in any of the biomechanical measurements among both systems. However, in terms of posterior bending, the single Y plating group exhibited the significantly lowest construct stiffness, failure stiffness and failure load. The differences between the two plating groups (PP and PTP) were insignificant, with the exception that failure stiffness was significantly higher in the posterior (PTP) group than in the parallel (PP) group. After the 5000-cycle loading, no intercondylar segment separation or screw loosening occurred in any of the fixation system, regardless of loading direction. Besides, we found that failure loads were positively correlated with BMD in the parallel group.

Successful treatment for comminuted DHFs requires bicolumnar fixation with sufficient biomechanical stability [7, 8] to allow early elbow range of motion and avoid post-operative complications such as non-union and implant failure [4–6]. Although several studies had found that parallel plating can provide good clinical results and is biomechanical superior to orthogonal plating in osteoporotic fracture [1, 22], it is associated with a greater extent of soft tissue dissection in the distal humerus [11, 12] a higher HO formation rate [23, 24], and greater difficulty in contouring and applying the lateral plates [19]. Posterior two-plate fixation is considered to be an alternative technique [17] that also has good functional outcomes, high union rates, and low complication rates in c-type DHFs [16]. The biomechanical performance for posterior plating in c-type DHFs is not clear. Previously, one biomechanical study analyzed the application of posterior and orthogonal two plating for a supracondylar type (a-type) DHF model using either two locking plates (LP) or two non-locking plates (NLP) [17]. The study showed that LP group was not superior to NLP group if plates were applied in the same direction. However, the construct stiffness may be higher when locking plates were applied orthogonally than those applied dorsally [17]. In the present study, we compared the posterior two plates system with the parallel two plates system in axial and sagittal loading. We found out that although the posterior configuration showed comparable construct stiffness and failure strength to the parallel one, failure modes were different, occurring in the form of construct breakage and bone fracture in the parallel group and plastic deformation in the posterior group. During the fatigue loading test, most of the posterior plates continued to bend in the opposite direction until it reached the amount of displacement that met the definition of load-to-failure. This finding helps us to realize that the posterior buttress effect of the plate should have an influence on the strength of the posterior fixation. Moreover, no implant failure and loosening occurred after the fatigue test in the posterior systems. Thus, as with other promising clinical result on posterior fixation for c-type DHFs [16], our study provides a biomechanical basis for the use of posterior two plating in treating comminuted DHFs as seen in the parallel plating to sustain the flexion and extension loading force encountered during early elbow range of motion exercise [18, 25]. However, additional interfragmentary screws are required to fix the intercondylar fragments when posterior plates were used. Although a larger approach on one side of the distal humerus is required for interfragmentary screw fixations, it should be still a smaller approach than that needed for parallel double plating.

Clinically, a non-locking single posterior Y plate has also been used to treat supracondylar [14, 26] and intracondylar DHFs [14, 15, 27, 28] successfully with promising results such as high union rate [26, 29], high satisfaction rate [14], high functional scores [14, 15] and low loosening rate [15]. When applying a traditional Y plate, surgeons have to curve the arms to “envelope” the epicondyles and add transverse screws or small k-wires for intercondylar fracture fixation [14]. Biomechanically, stress is distributed more equally along both columns when using a Y plate, and it allows easier epiphyseal screw fixation as compared to the traditional two-plate constructs that require additional intercondylar screw fixation [30]. Only a limited number of biomechanical studies (1 supracondylar model [31] and 2 intercondylar models [28, 30]) have compared the traditional Y-plate construct (screw trajectory from only antero-posterior direction without intercondylar fixation) with the orthogonal configuration, for which results were inconsistent. In addition, there is a lack of comparison of intercondylar DHFs fixation of Y-plating with other two types of double plating (posterior and parallel). Here, we used a pre-contoured Y plate with two opposite distal plate arms to treat DHFs, which a technique resembling interdigitation of both column screws in parallel construct is applied (Fig. 1d-e) to allow direct intercondylar fixation from each arm of the plate. In the present study, we found several biomechanical features regarding the Y plate fixation for c-type DHFs. First, the failure mode is all plate plastic deformation in the opposite direction of the loading force. Second, a single posterior Y plate construct is biomechanically weaker than other two-plate constructs. Some biomechanical studies that compared single Y plating with orthogonal two plating for DHF fixation [28, 30, 31] showed that the single Y plating is weaker than orthogonal two plating in sagittal [30, 31] and axial [28] plane. Our result also showed that a single Y plating is biomechanically weaker in the sagittal direction, but the strength was not inferior to that of the parallel and posterior two plate constructs in the axial loading direction. Although there was also no intercondylar fixation failure for Y plate fixation after the fatigue testing, a force limitation is still suggested when patient is engaged in early elbow flexion-extension exercise, as it is biomechanically weaker.

Different configurations may lead to different failure modes since mechanical failure is affected by loading directions, the number and orientation of plates/screws in peri-articular bone and BMD [11, 20, 32]. The findings of the current study and those of other studies suggest that failure modes for the parallel construct tend to be more catastrophic as compared to other configurations, which may be caused by the distal/proximal screw pulling out [11, 18, 25] or proximal bone fractures [7, 11, 18, 25]. The failure modes of posterior plating system in c-type DHFs have not been revealed before in the literature. In present study, we found that failure modes occurred mostly in the form of implant plastic deformation (plate bending) for either single or double posterior plating. The results provided us with valuable insights that the posterior plate(s) should serve as buttress plating during axial and sagittal force application, as has been observed in volar or dorsal buttress plate bending in comminuted distal radius fracture models [33, 34].

Moreover, we found that failure strength is positively correlated with BMD in the parallel constructs but not in the posterior constructs. The positive correlation may infer that when the bone quality improves, the strength leading to bone-screw loosening in the parallel construct may also increase. In comparison, as most posterior plates bend continuously and reach the failure threshold before implant breakage, the failure strength should rely more on the material stiffness rather than on the specimen BMD. Thus, it appears reasonable to propose that stiffer plates may have stronger biomechanical properties in posterior plating. However, future studies are required to confirm this hypothesis.

There are some limitations in this biomechanical study. First, the number of specimens is small, especially in the axial groups. Secondly, the clinical results and the condition of the soft tissue surrounding the elbow could not be evaluated biomechanically. The pre-contoured Y-plating may require more soft tissue dissection than the traditional Y plate, as the distal plate ends extend and cover both epicondyles. In addition, the application of interfragmentary screws during posterior plate fixation may require a larger soft tissue dissection, at least, on one side of the epicondyles. However, through standardized testing protocol, we were able to compare biomechanical strength in a more consistent way and thus could provide useful information for clinical applications. Third, we performed mechanical testing in axial and posterior bending directions. Varus bending and torsional loads were not tested. Although varus bending moments may occur over elbows when arms are abducted, studies has shown that the greatest forces across elbow joints occurs in daily activity were in the sagittal and axial directions [35]. Torsional loads were performed in previous studies [32, 36], but there were no sufficient literature references suggesting that torsional forces may lead to clinical failure [18]. Thus, the main experiment protocol of ours and other biomechanical studies [8, 11, 18–21, 28, 37] focused on mechanical testing in the axial and sagittal plane without testing the varus and torsional loads. Besides, it is recommended that early varus bending and torsional force should be avoided during early controlled elbow flexion and extension exercise to avoid early fixation failure for DHFs [12, 38]. Fourth, although we measured the intercondylar displacements and interfragmental medial and lateral column displacements, the parameters of angular deformation between the distal and the shaft fragments in different loading directions were not recorded in the present study.

## Conclusion

We found that when using two plates for treating comminuted DHFs, there were no significant differences in terms of most biomechanical measurements between posterior and parallel fixation. However, the single pre-contoured posterior Y plate construct was biomechanically weaker in the sagittal plane than the parallel and the posterior two-plate constructs, although there was no fixation failure after the fatigue test for all groups regardless of the fixation methods.

## Supplementary Information

**Additional file 1: Supplementary Table 1.** Post-hoc Mann-Whitney test between the parallel, Y, and posterior two plating constructs (three tests for pairwise comparisons of 3 groups) for different biomechanical parameters after cyclic loading.

## Data Availability

The datasets used and/or analysed during the current study are available from the corresponding author on reasonable request.
