# Supplementary material for: Does posterior configuration have similar strength as parallel configuration for treating comminuted distal humerus fractures? A cadaveric biomechanical study
Source: BMC Musculoskelet Disord. 2021 May 14;22:440. doi: 10.1186/s12891-021-04302-9 (PMC8122534; doi:10.1186/s12891-021-04302-9)
Supplement: Supplementary file 1 — Additional file 1: Supplementary Table 1. Post-hoc Mann-Whitney test between the parallel, Y, and posterior two plating constructs (three tests for pairwise comparisons of 3 groups) for different biomechanical parameters after cyclic loading. [file 12891_2021_4302_MOESM1_ESM.docx]

**Supplementary Table 1.** Post-hoc Mann-Whitney test between the parallel, Y, and posterior two plating constructs (three tests for pairwise comparisons of 3 groups) for different biomechanical parameters after cyclic loading.

|  | PP versus YP |  | PP versus PTP |  | YP versus PTP |  |
| --- | --- | --- | --- | --- | --- | --- |
|  | Axial loading (*P*-value) | | | | |  |
| Stiffness (50-200N)(N/mm) | 0.886 |  | 1.000 |  | 1.000 |  |
| Elastic limit (N) | 0.343 |  | 0.886 |  | 0.486 |  |
| Failure stiffness (N/mm) | 0.486 |  | 1.000 |  | 0.486 |  |
| Failure load (N) | 0.114 |  | 0.114 |  | 0.686 |  |
| Lateral column displacement (mm) | 1.000 |  | 0.486 |  | 0.486 |  |
| Medial column displacement (mm) | 0.343 |  | 0.886 |  | 0.057 |  |
| ΔIntercondylar width (mm) | 0.343 |  | 0.486 |  | 0.200 |  |
|  |  |  |  |  |  |  |
|  | PP versus YP |  | PP versus PTP |  | YP versus PTP |  |
|  | Posterior bending (*P*-value) | | | | | |
| Stiffness (50-200N)(N/mm) | 0.008** |  | 0.056 |  | 0.008** |  |
| Elastic limit (N) | 0.222 |  | 1.000 |  | 0.841 |  |
| Failure stiffness (N/mm) | 0.008** |  | 0.016* |  | 0.008** |  |
| Failure load (N) | 0.008** |  | 0.691 |  | 0.008** |  |
| Lateral column displacement (mm) | 0.008** |  | 0.421 |  | 0.008** |  |
| Medial column displacement (mm) | 0.008** |  | 0.841 |  | 0.008** |  |
| ΔIntercondylar width (mm) | 0.222 |  | 0.841 |  | 0.095 |  |

PP: parallel plate group; YP: Y plate group; PTP: posterior two plate group

Post-hoc Mann-Whitney test: **p*<0.05; ** *p*<0.01
